# Early intervention for young children with autism spectrum disorder: protocol for a scoping review of economic evaluations

**DOI:** 10.1186/s13643-021-01847-7

**Published:** 2021-11-10

**Authors:** Katherine Pye, Hannah Jackson, Teresa Iacono, Alan Shiell

**Affiliations:** 1 School of Psychology & Public Health, La Trobe University, Bundoora, Australia; 2 La Trobe Rural Health School, La Trobe University, Bendigo, Australia

**Keywords:** Economic evaluation, Autism, Early intervention, Scoping review

## Abstract

**Background:**

In many countries, children who are diagnosed with autism during the first 5 years of life are offered a range of early intervention options. These options vary considerably in the theoretical approaches and techniques applied, their intensity and duration, settings, the person/s delivering supports and the training they require. Early interventions are a significant contributor to total autism-related costs in Western countries, but only in the last 10–20 years has there been adequate outcome data to enable the comparison of different interventions’ cost-effectiveness. This protocol describes a scoping review to better understand what economic evaluations have been completed in this field, and the methods used to date.

**Methods:**

We will systematically search the following databases from their inception to 2021 for eligible studies: MEDLINE, EMBASE, PsycINFO, Econlit, PEDE, NHS EED and HTA. Full economic evaluations of any types of early intervention for children with autism prior to school entry will be included. Two reviewers will screen the studies, extract the data and assess the study quality using established checklists. The risk of bias will be assessed using the extended CHEC-list for all studies and, additionally, the Philips checklist for modelled studies. Quality of reporting will be assessed using the CHEERS checklist. A narrative synthesis will be completed to collate the findings, describe the methods used and identify which interventions have been researched from an economic perspective.

**Discussion:**

This review will provide researchers, policymakers and service providers with current information about the economic evidence for early interventions for young children with autism and point to priorities for further research. It will inform future economic evaluations by highlighting the gaps or inconsistencies in the methods used to date. Limitations of the review will be acknowledged and discussed.

**Systematic review registration:**

Open Science Framework: https://osf.io/sj7kt

**Supplementary Information:**

The online version contains supplementary material available at 10.1186/s13643-021-01847-7.

## Background

### Rationale

Individuals with autism demonstrate many strengths, while also experiencing a range of challenges. Diagnostic features include social communication difficulties that impact the development and maintenance of relationships, alongside repetitive and/or restricted patterns of behaviour [1]. People with a diagnosis of autism spectrum disorder (referred to herein as autism) vary greatly in their core abilities and levels of functioning in daily life. They can often experience other conditions, such as intellectual disability, mood disorders, anxiety or attention-deficit/hyperactivity disorder (ADHD). Naturally, individual aspirations, needs and outcomes vary greatly.

Recent epidemiological studies indicate that as many as 1 in 54 children have a diagnosis of autism at the age of 8 in the USA [2]. Reported prevalence figures have risen in recent decades in the USA [2], UK [3] and Australia [4], with calls for better surveillance across Europe [5] and China [6]. In Australia, participants with a primary diagnosis of autism make up approximately 29% of the National Disability Insurance Scheme: the largest single diagnostic group [7]. In the USA, an estimated $309,873,907 was invested in autism research in 2014, 81% of which came from the federal government [8]. Buescher and colleagues reviewed the UK and US literature, collating data relating to autism prevalence, level of functioning and residence [9]. They combined these data with mean support costs, opportunity costs and productivity losses and concluded that childhood autism costs amounted to £3.4 billion per year in the UK and US$66 billion per year in the USA. The largest contributors were direct nonmedical costs, including early intervention. In Australia, annual total costs in 2010, including impact on quality of life, were estimated at approximately AUD$9.7 billion or $87,000 per person per year [10].

Early intervention has recently been defined as “a collection of clinical techniques, applied in combination, which aims to support the acquisition of developmental or educational skills, in order to promote well-being and community participation” [11]. The Early Childhood Intervention Association in Victoria, Australia, describes early (childhood) intervention as “the process of providing specialised support and services for infants and young children with disability and/or developmental delay, and their families in order to promote development, well-being and community participation” [12]. Implicit in both of these definitions is that intervention is additional to the usual care offered to young children, such as mainstream early childhood education or childcare, and that goal-oriented techniques are consciously applied in intervention, as opposed to incidental benefits that may come from a child’s situation or experience.

Typically, early intervention might include therapy delivered by professionals from such disciplines as speech therapy, psychology or occupational therapy, or more comprehensive approaches that target a wide range of developmental domains, such as the Early Start Denver Model [13] or Applied Behaviour Analysis [14]. However, there are many alternative options available to families, including therapies based on the use of animals or technology, and various parent training programmes. Each intervention can be delivered at different intensities, and most can also be adapted for delivery in different settings, by people in different roles, or with individuals or groups of children. Each model therefore comes with different financial, time and other costs.

Despite the diversity in early intervention, there is some cohesion in the aims of these supports, namely, to promote skill development in order to improve well-being and participation in the community, in the short and long term. Whitehouse and colleagues recently completed an umbrella review of the effectiveness research in this field, including 58 systematic reviews published since 2010 [11]. There is a body of evidence to support a range of behavioural and developmental approaches, some of which have more recently been classified as “naturalistic, developmental and behavioural interventions”. Despite this body of research, the question remains: which interventions provide better value to the children, families and broader society investing in them?

The ultimate objective, improved quality of life, might encompass increased social and economic participation, reduced carer burden and reduced need for educational, social and other supports later in life. These outcomes are of economic interest as families, policymakers and support providers endeavour to use limited resources (particularly time and effort) for optimal gains (namely, child skill acquisition, child and family participation and well-being). Child outcomes have most often been measured with a narrow focus on cognitive ability, language skills and adaptive behaviour [14, 15]. There have been calls for validated tools to measure broader functional outcomes such as social participation, employment and health-related quality of life [16–18].

Economic evaluations are increasingly being used to inform the allocation of limited healthcare resources [19]. The wide-ranging methods of providing supports to children and their families naturally incur wide-ranging costs, and the consequences of early intervention for the child, their family and community in the short and long terms are increasingly better understood. Economic evaluations may involve the application of a range of assumptions, perspectives, time horizons, costs and consequences; their findings are likely to differ widely as a result. This presents an opportunity to explore those variables and their possible impact on cost-effectiveness.

Weinmann and colleagues reviewed behavioural interventions in 2009 and concluded that cost and outcome data available at that time was inadequate for economic analysis [20]. More recently, researchers commissioned by the UK’s National Institute for Health Research published a health technology assessment [15], including a review of the cost-effectiveness of early intensive behavioural interventions. Their search of the literature, from database inception to November 2017, yielded six economic evaluations of this type of intervention for their analysis. Most recently, Sampaio and colleagues completed a systematic review of economic evaluations of pharmacological or psychosocial/behavioural treatments for attention-deficit/hyperactivity disorder (ADHD) or autism in children and adolescents, with the aim to provide an overview of recent evidence in the field. Their search of literature from 2010 to 2020 yielded two studies relating to autism.

The current study will extend the foci of these prior reviews by implementing a broader search that will include a wider array of interventions to determine what, if any, economic evidence exists for them, and to describe and critique the methods used. The intention is not to draw a conclusion about a particular type of intervention or method, but to explore the field and understand the range of evidence that has been provided to date.

Prior reviews have demonstrated that economic evaluations in this field have been scarce, despite the number of interventions and supports available and the resources used to provide them. There is no established method for valuing the outcomes of interventions accessed by autistic children, and previous reviewers have criticised studies that have taken a cost minimisation approach or neglected to consider spillover effects on families [15]. In preparation for future economic evaluations, we seek to understand approaches that have been taken previously, to identify which areas are understudied and which, if any, evidence has been well-established.

Indications for scoping reviews, rather than systematic reviews, include to identify the types of evidence and research methods applied in a given field, rather than to directly inform decision-making by, for example, determining the effectiveness of a specific programme [21, 22]. Given the exploratory nature of this review and the heterogeneity in the included interventions, a scoping review will be conducted.

### Objectives

The following research questions will be addressed: what economic evidence is there for early interventions aimed at young children with autism, and how have researchers evaluated their costs and benefits to date? The specific aims of this review are as follows:

1. To collate the best available information about the economic efficiency of interventions for autistic children during the years prior to starting school
2. To examine the methods used in conducting economic evaluations of early interventions for young children with autism
3. To understand the extent to which different types of intervention have been evaluated economically and identify where gaps exist in the evidence
4. To critique the quality of the available economic evidence
5. To explore how and why relative cost-effectiveness varies across settings

## Methods

The review protocol has been registered within the Open Science Framework database (osf.io/sj7kt) and is being reported in accordance with the reporting guidance provided in the Preferred Reporting Items for Systematic Reviews and Meta-Analyses Protocols (PRISMA-P) statement [23] (see Additional file 1).

### Inclusion criteria

The inclusion criteria are described below in terms of participants, concept and context, as recommended in scoping reviews [24].

*Participants* of included studies will be children with a diagnosis of autism, or considered to have increased likelihood of autism, up until the age of school entry in the study location. Studies covering a broader population will be included if disaggregated data are provided for the preschool autistic subgroup. Formal diagnoses may include autism spectrum disorder (DSM-5 or ICD-11) or previously used diagnostic terms (e.g. autistic disorder).

The core *concepts* of this review are (a) economic evidence related to (b) any interventions for the population described above. Included studies will be full economic evaluations (i.e. cost-consequence, cost-effectiveness, cost-utility or cost-benefit analyses) comparing the costs and consequences of two or more programmes, one of which must be a form of early intervention [25]. Studies evaluating intervention costs or outcomes alone will not be included.

Intervention of any kind is defined for the purposes of this review as a modification or addition to standard care that is implemented with the intention of improving the well-being of an autistic child and/or their family. This includes clinical techniques, environmental modifications, pharmaceuticals, the use of therapy animals, and programmes to identify autistic children earlier as a means to access better/earlier supports. No specific settings or delivery modes will be excluded, and all comparators will be included. Studies must include outcomes that relate to the child with autism and/or their family. Studies that include only outcomes relating to people outside the family (e.g. educators) will be excluded.

The *context* of this review will be open: there are no limitations to country, setting or culture. Evaluations may be based on trial or modelled data, or combinations of both. Included studies will be published with full text in English due to resource limitations. No restriction will be placed on publication date as we seek to understand the breadth of economic research, including the full array of methods used to value the costs and benefits of programmes.

### Information sources

Studies will be obtained from the following research databases: MEDLINE, EMBASE, PsycINFO and EconLit. Secondary searches will be completed in other economic evaluation databases, including the National Health Service Economic Evaluations Database (NHS EED) and Health Technology Assessments, both via the University of York’s Centre for Reviews and Dissemination (CRD) Database, as well as the Pediatric Economic Database Evaluation (PEDE). Grey literature will be identified by searching Google, ProQuest Dissertations and Theses Global, the New York Academy of Medicine Grey Literature Report and the ISPOR Presentations Database. All sources will be searched from their inception onwards. Systematic reviews of economic evaluations will not be included in the analysis but will be searched for relevant primary studies and forward citations (using Google Scholar). The reference lists and forward citations of eligible studies will also be searched.

### Search strategy

A search strategy was developed in MEDLINE in consultation with two research advisors from La Trobe University’s library who are experienced in systematic searches (see Additional file 2). This strategy was reviewed by a research advisor using the CADTH PRESS checklist [26] and will be translated for advanced searching in the other primary databases listed above.

### Data management

Search results will be imported into Covidence [27], where duplicates will be automatically removed. Screening, data extraction and quality assessments are expected to be completed using the Covidence platform.

### Selection process

Two researchers (KP and HJ) will each independently screen all titles and abstracts in stage 1, thereby excluding any studies that are clearly irrelevant to this review. Full texts will then be screened by the same two reviewers in stage 2, again independently. Any disagreement will be resolved through discussion and consensus involving a third reviewer (AS) when necessary.

The results of the search and selection process will be reported in full and presented in a Preferred Reporting Items for Systematic Reviews and Meta-Analyses (PRISMA) flow diagram [28, 29].

### Data extraction

Data will be extracted using Covidence Data Extraction 2.0 [27]. This allows reviewers to customise a data extraction template within the reviewing platform. The template will be piloted and refined by the lead researcher (KP) before two reviewers embark on the bulk of reviews. Disagreements will again be resolved through consensus and discussion with a third reviewer (AS) when required. Study authors will be contacted by email (maximum of three attempts) for clarification or more detailed data when required.

Reviewers will not be blinded to study authors or titles during data extraction, in order to facilitate the identification of overlapping reports.

### Data items

The following data are expected to be extracted from included studies:

**Table:** 

**Study characteristics**	**Study methods and outcomes**
First author Country Year of publication Type of intervention/s Comparator/s Population diagnosis/es Eligibility criteria Number of study participants Age of children Perspective Type of EE Analytic approach (trial vs modelled/mixed)	Model structure Time horizon Discount rate (costs and effects) Currency Reference year of analysis Assumptions made Types of resources identified Source/s of resource use data Cost figures Types of consequences identified (positive and adverse) Source/s of consequences data, including outcome measures used Value of consequences ICERs Uncertainty analysis methods Outcomes of uncertainty analysis Conclusions

Monetary data will be adjusted for inflation and converted to a common currency (USD) to facilitate the comparison of the results. Disaggregated data, such as unit costs or separate wages and equipment costs, will be recorded where available.

### Analysis

Of particular interest to this scoping review are the types of outcomes assessed and the methods used to identify, measure and value these outcomes. A narrative synthesis of the features and findings of included studies, including factors most impacting results, will be produced to address this review’s aims. The results will be presented visually where appropriate.

### Critical appraisal

In order to collate the best available evidence in a field, we must be cognisant of the methodological and reporting quality of the evidence considered. Several checklists have been recommended to support the appraisal of study quality. The health technology assessment checklist by Phillips et al. [30] has been recommended by the Joanna Briggs Institute [24] and the National Institute for Health and Care Excellence [31] for reviewing the modelled economic evaluations. The Consensus on Health Economic Criteria (CHEC) list has been recommended for trial-based evaluations but is arguably inadequate for modelled studies on its own [32]. Two reviewers will therefore assess the risk of bias of all studies using one well-established tool, the Consensus on Health Economic Criteria (CHEC)-extended checklist [33, 34]. Given the small number of anticipated studies, the risk of bias in modelled studies will be further assessed with the Philips Checklist [30], as recommended by van Mastrigt and colleagues [35]. The quality of reporting will be assessed by two reviewers using the Consolidated Health Economic Evaluation Reporting Standards (CHEERS) checklist [36].

## Discussion

This review will inform future economic research in the field of autism in childhood, and potentially other areas of child development more broadly. To this end, gaps and inconsistencies in the research to date will be summarised and discussed, and the methods used to measure, value and compare costs and consequences will be described and appraised. This information will help researchers to first ascertain whether or not an economic evaluation is required in their field of interest, and then to consider a range of possible methods that might be applied. The findings of this review will provide policymakers and service providers with information about the quality of economic evidence that is currently available to them in this field.

There are several anticipated limitations of this review. Studies of the effectiveness of interventions for children on the autism spectrum have generally been regarded as of low-moderate quality, due largely to the risk of bias and lack of reporting of adverse effects [11, 15]. Economic evaluations included in this review are therefore likely to be limited by this uncertainty, and we expect sensitivity analysis in each original study to reflect this uncertainty. Comparators are likely to include a “treatment as usual” option, which could vary considerably between settings and studies.

In any review of economic evaluations, direct comparison of study findings is inherently difficult given that costs, policies and possibly even consequences are setting specific. This review will not provide synthesised findings from included studies, but we will be comparing the methods that they have used and will consider the impact any methodological differences may have had on findings, to inform future evaluations in this field.

Any future amendments to this protocol will be briefly updated in the OSF registration and clearly described in the final manuscript. The results of the scoping review will be disseminated via publication in a peer-reviewed journal and conference presentations.

## Supplementary Information

**Additional file 1.** PRISMA-P 2015 Checklist.

**Additional file 2.** MEDLINE search strategy.

## Data Availability

There is no data applicable to the current protocol. The data extracted and analysed in the prospective review will be made available from the corresponding author on request.
