# Supplementary material for: Early intervention for young children with autism spectrum disorder: protocol for a scoping review of economic evaluations
Source: Syst Rev. 2021 Nov 10;10:295. doi: 10.1186/s13643-021-01847-7 (PMC8579569; doi:10.1186/s13643-021-01847-7)
Supplement: Supplementary file 2 — Additional file 2. MEDLINE search strategy. [file 13643_2021_1847_MOESM2_ESM.docx]

## Additional File 2

#### Search strategy: MEDLINE

1. economics/
2. exp “costs and cost analysis”/
3. economics, medical/
4. economics, nursing/
5. economics, pharmaceutical/
6. (economic$ or cost$ or pharmacoeconomic$ or pharmaco-economic$).ti,ab.
7. expenditure$.ti,ab.
8. (value adj2 money).ti,ab.
9. budget$.ti,ab.
10. economics, dental/
11. exp economics, hospital/
12. 1 or 2 or 3 or 4 or 5 or 6 or 7 or 8 or 9 or 10 or 11
13. exp child/ or exp infant/
14. child$.ti,ab.
15. preschool$.ti,ab.
16. pre-school$.ti,ab.
17. toddler$.ti,ab.
18. kindergarten$.ti,ab.
19. exp Child Day Care Centers/
20. Child Care/
21. Schools, nursery/
22. (childcare or child-care or child care).ti,ab.
23. nursery school$.ti,ab.
24. (child$ adj2 (day care or daycare)).ti,ab.
25. 13 or 14 or 15 or 16 or 17 or 18 or 19 or 20 or 21 or 22 or 23 or 24
26. exp autism spectrum disorder/
27. (autis$ or ASD or ASDs or ASC or AAC).ti,ab.
28. Asperger$.ti,ab.
29. autism.ti,ab.
30. 26 or 27 or 28 or 29
31. 12 and 25 and 30
